# *endo*-3,3-Dimethyl-4-oxobicyclo­[3.1.0]hexan-2-yl methane­sulfonate

**DOI:** 10.1107/S1600536810010901

**Published:** 2010-03-27

**Authors:** Adrian Kremer, Bernadette Norberg, Alain Krief, Johan Wouters

**Affiliations:** a Department of Chemistry, University of Namur, 61 Rue de Bruxelles, B-5000 Namur, Belgium

## Abstract

The relative configuration of the *endo* isomer of the title compound, C_9_H_14_O_4_S, has been established and the conformation of the diastereoisomer is discussed. The five-membered ring adopts an envelope conformation. The conformation of the methane­sulfonate substituent is stabilized by inter­molecular C—H⋯O hydrogen bonds. The crystal packing results in alternating layers of polar methane­sulfonates and stacked bicyclo­hexa­nyl rings parallel to *ab*.

## Related literature

For related enanti­oselective syntheses, see: Krief (1994 ▶); Krief *et al.* (2000 ▶). For puckering parameters and theoretical torsion angles, see: Cremer & Pople (1975 ▶); Dunitz (1979 ▶).
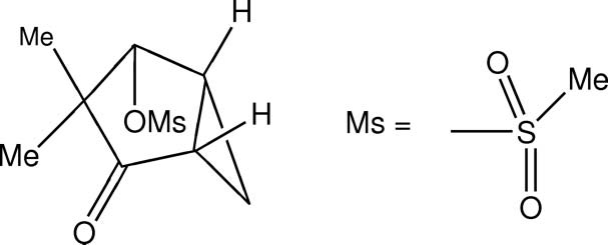


## Experimental

### 

#### Crystal data

C_9_H_14_O_4_S
*M* *_r_* = 218.27
Triclinic, 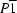

*a* = 5.8558 (3) Å
*b* = 7.7497 (4) Å
*c* = 12.2527 (6) Å
α = 84.290 (4)°
β = 79.531 (4)°
γ = 72.070 (5)°
*V* = 519.66 (5) Å^3^
*Z* = 2
Mo *K*α radiation
μ = 0.30 mm^−1^
*T* = 293 K
0.35 × 0.14 × 0.12 mm

#### Data collection

Oxford Diffraction Xcalibur diffractometer with a Ruby (Gemini ultra Mo) detector
Absorption correction: multi-scan (*CrysAlis PRO*; Oxford Diffraction, 2009 ▶) *T*_min_ = 0.904, *T*_max_ = 0.966
6128 measured reflections
3432 independent reflections
2283 reflections with *I* > 2σ(*I*)
*R*_int_ = 0.019

#### Refinement

*R*[*F* ^2^ > 2σ(*F* ^2^)] = 0.046
*wR*(*F* ^2^) = 0.130
*S* = 0.99
3432 reflections
130 parameters
H-atom parameters constrained
Δρ_max_ = 0.36 e Å^−3^
Δρ_min_ = −0.31 e Å^−3^

### 

Data collection: *CrysAlis PRO* (Oxford Diffraction, 2009 ▶); cell refinement: *CrysAlis PRO*; data reduction: *CrysAlis PRO*; program(s) used to solve structure: *SHELXS97* (Sheldrick, 2008 ▶); program(s) used to refine structure: *SHELXL97* (Sheldrick, 2008 ▶); molecular graphics: *ORTEPIII* (Burnett & Johnson, 1996 ▶) and *PLATON* (Spek, 2009 ▶); software used to prepare material for publication: *SHELXL97* .

## Supplementary Material

Crystal structure: contains datablocks I, global. DOI: 10.1107/S1600536810010901/dn2550sup1.cif

Structure factors: contains datablocks I. DOI: 10.1107/S1600536810010901/dn2550Isup2.hkl

Additional supplementary materials:  crystallographic information; 3D view; checkCIF report

## Figures and Tables

**Table 1:** Hydrogen-bond geometry (Å, °)

*D*—H⋯*A*	*D*—H	H⋯*A*	*D*⋯*A*	*D*—H⋯*A*
C4—H4⋯O4^i^	0.98	2.48	3.302 (4)	141
C9—H9*B*⋯O2^ii^	0.96	2.54	3.485 (2)	169

Symmetry codes: (i) 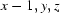; (ii) 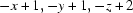.

**Fig. 1.**
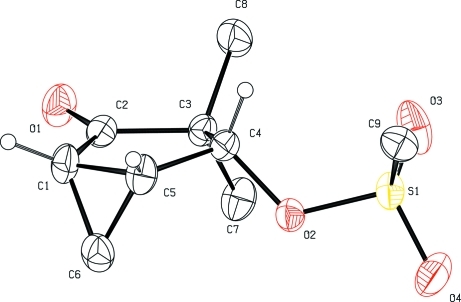
Conformation (ORTEP view) of the title compound. Only H atoms on chiral carbons have been retained for clarity. Displacement ellipsoids for non-H atoms are drawn at the 30% probability level.

## supplementary crystallographic information

### Comment

In the course of a work involving the enantioselective synthesis of didesmethyl-deltametrinic acid (Krief *et al.*, 2000; Krief, 1994) both the *exo* and *endo* isomers of 3,3-dimethyl-4-oxobicyclo[3.1.0]hexan-2-yl methanesulfonate were synthesized and characterized.

The X-ray crystallography study reported here determined the relative stereochemistry of the *endo* diasteroisomer : C(1) *S*, C(4) *R*, and C(5) *R*. The compound crystallizing in a centrosymetric space group, one obtains the racemic mixture *S*,*R*,*R*/*R*,*S*,*S*.

The five-membered ring C1—C5 adopts an envelope conformation. Puckering parameter Phi is 260.1 (8)° and close to the expected value of k *x* 36° (Cremer & Pople, 1975), suggesting that the presence of a *sp*^2^ carbon (C2) in the five-membered ring does not significantly distort its conformation. The observed values of torsion angles defining the C1—C5 ring (Table 1) fairly well follow the theoretical sequence of torsion angles -ω1, ω2, -ω2, ω1 and 0 (Dunitz, 1979) characteristic of an envelope conformation.

Atom C6 of the fused three-membered ring deviates by +1.250 (2) Å from the mean plane defined by the five atoms of the C1—C5 ring (Figure 1).

Steric effects resulting from C6 being in *cis* of the mesylate substituent on O2, constrain the conformation of the methanesulfonate group. Positions of the oxygen atoms O3 and O4 of the sulfonate group are further explained by intra and intermolecular CH···O hydrogen bondings. Indeed O3 forms an intramolecular H bond [O3···H4 = 2.81 Å] with H4 of C4 that carries the mesylate. An intermolecular H bond with H4 further involves O4 [C(4)—H4 ··· O4*_i_*: D···A = 3.302 (4) Å; H···A = 2.48 Å; D - H···A = 141°, *i* = *x*-1,*y*,*z*].

Packing is also reinforced by van der Waals interactions resulting in alterning layers of polar methanesulfonates and stacked bicyclohexanyl rings parallel to the *ab* cell planes.

### Experimental

Synthesis of the compound will be detailed elsewhere.

Crystals were obtained by evaporation at 5°C of solutions in diethylether.

### Refinement

All H atoms were placed at idealized positions and allowed to ride on their parent atoms, with C—H = 0.97 Å and *U*_iso_(H) = 1.2*U*_eq_(C) for methylene groups and *U*_iso_(H) = 1.5*U*_eq_(C) for the methyl group.

### Crystal data

**Table:**

C_9_H_14_O_4_S	*Z* = 2
*M**_r_* = 218.27	*F*(000) = 232
Triclinic, *P*1	*D*_x_ = 1.395 Mg m^−^^3^
Hall symbol: -P 1	Mo *K*α radiation, λ = 0.71073 Å
*a* = 5.8558 (3) Å	Cell parameters from 2686 reflections
*b* = 7.7497 (4) Å	θ = 3.2–32.6°
*c* = 12.2527 (6) Å	µ = 0.30 mm^−^^1^
α = 84.290 (4)°	*T* = 293 K
β = 79.531 (4)°	Prism, colorless
γ = 72.070 (5)°	0.35 × 0.14 × 0.12 mm
*V* = 519.66 (5) Å^3^	

### Data collection

**Table:**

Oxford Diffraction Xcalibur diffractometer with a Ruby (Gemini ultra Mo) detector	3432 independent reflections
Radiation source: fine-focus sealed tube	2283 reflections with *I* > 2σ(*I*)
graphite	*R*_int_ = 0.019
Detector resolution: 10.3712 pixels mm^-1^	θ_max_ = 32.6°, θ_min_ = 3.2°
ω scans	*h* = −8→8
Absorption correction: multi-scan (*CrysAlis PRO*; Oxford Diffraction, 2009)	*k* = −8→11
*T*_min_ = 0.904, *T*_max_ = 0.966	*l* = −18→18
6128 measured reflections	

### Refinement

**Table:**

Refinement on *F*^2^	Primary atom site location: structure-invariant direct methods
Least-squares matrix: full	Secondary atom site location: difference Fourier map
*R*[*F*^2^ > 2σ(*F*^2^)] = 0.046	Hydrogen site location: inferred from neighbouring sites
*wR*(*F*^2^) = 0.130	H-atom parameters constrained
*S* = 0.99	*w* = 1/[σ^2^(*F*_o_^2^) + (0.0721*P*)^2^] where *P* = (*F*_o_^2^ + 2*F*_c_^2^)/3
3432 reflections	(Δ/σ)_max_ < 0.001
130 parameters	Δρ_max_ = 0.36 e Å^−^^3^
0 restraints	Δρ_min_ = −0.31 e Å^−^^3^

### Special details

**Table:**

Geometry. All esds (except the esd in the dihedral angle between two l.s. planes) are estimated using the full covariance matrix. The cell esds are taken into account individually in the estimation of esds in distances, angles and torsion angles; correlations between esds in cell parameters are only used when they are defined by crystal symmetry. An approximate (isotropic) treatment of cell esds is used for estimating esds involving l.s. planes.
Refinement. Refinement of *F*^2^ against ALL reflections. The weighted *R*-factor wR and goodness of fit *S* are based on *F*^2^, conventional *R*-factors *R* are based on *F*, with *F* set to zero for negative *F*^2^. The threshold expression of *F*^2^ > σ(*F*^2^) is used only for calculating *R*-factors(gt) etc. and is not relevant to the choice of reflections for refinement. *R*-factors based on *F*^2^ are statistically about twice as large as those based on *F*, and *R*- factors based on ALL data will be even larger.

### Fractional atomic coordinates and isotropic or equivalent isotropic displacement parameters (Å^2^)

**Table:**

	*x*	*y*	*z*	*U*_iso_*/*U*_eq_	
C1	0.1819 (3)	0.4668 (2)	0.66360 (15)	0.0492 (4)	
H1	0.0668	0.4050	0.6508	0.059*	
C2	0.1965 (3)	0.6346 (2)	0.59887 (13)	0.0422 (3)	
C3	0.2668 (3)	0.75983 (19)	0.66753 (13)	0.0377 (3)	
C4	0.2405 (3)	0.6746 (2)	0.78727 (12)	0.0383 (3)	
H4	0.0950	0.7502	0.8323	0.046*	
C5	0.2143 (3)	0.4885 (2)	0.78131 (14)	0.0474 (4)	
H5	0.1187	0.4400	0.8439	0.057*	
C6	0.4088 (4)	0.3618 (2)	0.70955 (15)	0.0534 (4)	
H6A	0.4345	0.2330	0.7265	0.064*	
H6B	0.5558	0.3956	0.6814	0.064*	
C7	0.5212 (3)	0.7725 (3)	0.61787 (15)	0.0556 (5)	
H7A	0.6366	0.6539	0.6194	0.083*	
H7B	0.5646	0.8515	0.6606	0.083*	
H7C	0.5216	0.8202	0.5425	0.083*	
C8	0.0812 (4)	0.9504 (2)	0.66350 (19)	0.0642 (5)	
H8A	0.1226	1.0300	0.7066	0.096*	
H8B	−0.0787	0.9425	0.6935	0.096*	
H8C	0.0843	0.9972	0.5879	0.096*	
C9	0.2608 (3)	0.7738 (2)	1.04091 (14)	0.0503 (4)	
H9A	0.2548	0.8619	1.0920	0.075*	
H9B	0.3173	0.6540	1.0740	0.075*	
H9C	0.1012	0.7930	1.0235	0.075*	
O1	0.1502 (3)	0.67456 (18)	0.50596 (10)	0.0606 (4)	
O2	0.4535 (2)	0.64888 (14)	0.84223 (9)	0.0421 (3)	
O3	0.3691 (3)	0.97232 (17)	0.87030 (11)	0.0717 (4)	
O4	0.7018 (2)	0.7375 (2)	0.94357 (12)	0.0761 (5)	
S1	0.45905 (8)	0.79673 (6)	0.91953 (3)	0.04398 (14)	

### Atomic displacement parameters (Å^2^)

**Table:**

	*U*^11^	*U*^22^	*U*^33^	*U*^12^	*U*^13^	*U*^23^
C1	0.0598 (11)	0.0458 (9)	0.0546 (10)	−0.0288 (8)	−0.0180 (8)	−0.0019 (7)
C2	0.0405 (8)	0.0438 (8)	0.0452 (8)	−0.0140 (7)	−0.0116 (6)	−0.0025 (7)
C3	0.0408 (8)	0.0344 (7)	0.0426 (8)	−0.0156 (6)	−0.0133 (6)	0.0027 (6)
C4	0.0353 (7)	0.0398 (8)	0.0421 (8)	−0.0143 (6)	−0.0058 (6)	−0.0031 (6)
C5	0.0589 (10)	0.0487 (9)	0.0438 (9)	−0.0314 (8)	−0.0089 (7)	0.0059 (7)
C6	0.0680 (12)	0.0369 (8)	0.0601 (11)	−0.0172 (8)	−0.0211 (9)	0.0017 (7)
C7	0.0600 (11)	0.0673 (12)	0.0508 (10)	−0.0390 (10)	−0.0052 (8)	0.0036 (9)
C8	0.0767 (14)	0.0400 (9)	0.0783 (13)	−0.0074 (9)	−0.0359 (11)	−0.0025 (9)
C9	0.0526 (10)	0.0515 (10)	0.0414 (8)	−0.0116 (8)	0.0001 (7)	−0.0030 (7)
O1	0.0771 (9)	0.0638 (8)	0.0499 (7)	−0.0244 (7)	−0.0290 (6)	0.0015 (6)
O2	0.0476 (6)	0.0399 (6)	0.0409 (6)	−0.0116 (5)	−0.0131 (5)	−0.0061 (4)
O3	0.1261 (14)	0.0447 (7)	0.0544 (8)	−0.0425 (8)	−0.0113 (8)	0.0004 (6)
O4	0.0484 (8)	0.1248 (13)	0.0661 (9)	−0.0348 (8)	−0.0039 (6)	−0.0363 (9)
S1	0.0507 (3)	0.0491 (2)	0.0385 (2)	−0.02405 (19)	−0.00324 (16)	−0.00920 (16)

### Geometric parameters (Å, °)

**Table:**

C1—C2	1.472 (2)	C6—H6B	0.9700
C1—C6	1.503 (2)	C7—H7A	0.9600
C1—C5	1.521 (2)	C7—H7B	0.9600
C1—H1	0.9800	C7—H7C	0.9600
C2—O1	1.2066 (19)	C8—H8A	0.9600
C2—C3	1.534 (2)	C8—H8B	0.9600
C3—C7	1.532 (2)	C8—H8C	0.9600
C3—C8	1.543 (2)	C9—S1	1.7429 (17)
C3—C4	1.549 (2)	C9—H9A	0.9600
C4—O2	1.4747 (17)	C9—H9B	0.9600
C4—C5	1.506 (2)	C9—H9C	0.9600
C4—H4	0.9800	O2—S1	1.5687 (11)
C5—C6	1.467 (3)	O3—S1	1.4156 (14)
C5—H5	0.9800	O4—S1	1.4287 (14)
C6—H6A	0.9700		
			
C2—C1—C6	114.72 (14)	C1—C6—H6A	117.6
C2—C1—C5	107.13 (13)	C5—C6—H6B	117.6
C6—C1—C5	58.06 (11)	C1—C6—H6B	117.6
C2—C1—H1	120.3	H6A—C6—H6B	114.7
C6—C1—H1	120.3	C3—C7—H7A	109.5
C5—C1—H1	120.3	C3—C7—H7B	109.5
O1—C2—C1	125.52 (15)	H7A—C7—H7B	109.5
O1—C2—C3	123.47 (14)	C3—C7—H7C	109.5
C1—C2—C3	110.94 (13)	H7A—C7—H7C	109.5
C7—C3—C2	109.53 (13)	H7B—C7—H7C	109.5
C7—C3—C8	109.33 (15)	C3—C8—H8A	109.5
C2—C3—C8	108.62 (13)	C3—C8—H8B	109.5
C7—C3—C4	115.62 (13)	H8A—C8—H8B	109.5
C2—C3—C4	103.93 (11)	C3—C8—H8C	109.5
C8—C3—C4	109.53 (14)	H8A—C8—H8C	109.5
O2—C4—C5	106.44 (12)	H8B—C8—H8C	109.5
O2—C4—C3	113.84 (11)	S1—C9—H9A	109.5
C5—C4—C3	108.08 (12)	S1—C9—H9B	109.5
O2—C4—H4	109.5	H9A—C9—H9B	109.5
C5—C4—H4	109.5	S1—C9—H9C	109.5
C3—C4—H4	109.5	H9A—C9—H9C	109.5
C6—C5—C4	116.56 (15)	H9B—C9—H9C	109.5
C6—C5—C1	60.38 (11)	C4—O2—S1	120.23 (9)
C4—C5—C1	108.34 (13)	O3—S1—O4	119.56 (10)
C6—C5—H5	119.1	O3—S1—O2	110.12 (7)
C4—C5—H5	119.1	O4—S1—O2	103.74 (8)
C1—C5—H5	119.1	O3—S1—C9	108.61 (9)
C5—C6—C1	61.56 (12)	O4—S1—C9	108.62 (9)
C5—C6—H6A	117.6	O2—S1—C9	105.24 (8)
			
C6—C1—C2—O1	127.22 (19)	C8—C3—C4—C5	128.36 (15)
C5—C1—C2—O1	−170.65 (18)	O2—C4—C5—C6	−66.42 (17)
C6—C1—C2—C3	−55.74 (19)	C3—C4—C5—C6	56.24 (18)
C5—C1—C2—C3	6.39 (18)	O2—C4—C5—C1	−131.81 (14)
O1—C2—C3—C7	−70.5 (2)	C3—C4—C5—C1	−9.15 (18)
C1—C2—C3—C7	112.43 (15)	C2—C1—C5—C6	−108.87 (16)
O1—C2—C3—C8	48.9 (2)	C2—C1—C5—C4	1.83 (19)
C1—C2—C3—C8	−128.22 (16)	C6—C1—C5—C4	110.70 (16)
O1—C2—C3—C4	165.44 (17)	C4—C5—C6—C1	−96.92 (16)
C1—C2—C3—C4	−11.67 (17)	C2—C1—C6—C5	95.46 (16)
C7—C3—C4—O2	10.41 (18)	C5—C4—O2—S1	−146.30 (11)
C2—C3—C4—O2	130.47 (12)	C3—C4—O2—S1	94.74 (13)
C8—C3—C4—O2	−113.62 (15)	C4—O2—S1—O3	−42.98 (13)
C7—C3—C4—C5	−107.61 (15)	C4—O2—S1—O4	−172.07 (11)
C2—C3—C4—C5	12.45 (16)	C4—O2—S1—C9	73.90 (12)

### Hydrogen-bond geometry (Å, °)

**Table:**

*D*—H···*A*	*D*—H	H···*A*	*D*···*A*	*D*—H···*A*
C4—H4···O4^i^	0.98	2.48	3.302 (4)	141
C9—H9B···O2^ii^	0.96	2.54	3.485 (2)	169

Symmetry codes: (i) *x*−1, *y*, *z*; (ii) −*x*+1, −*y*+1, −*z*+2.

## References

[bb1] Burnett, M. N. & Johnson, C. K. (1996). *ORTEPIII* Report ORNL-6895. Oak Ridge National Laboratory, Tennessee, USA.

[bb2] Cremer, D. & Pople, J. A. (1975). *J. Am. Chem. Soc.* **97**, 1354–1358.

[bb3] Dunitz, J. (1979). * X-ray Analysis and the Structure of Organic Molecules*, p. 429, Ithaca: Cornell University Press.

[bb5] Krief, A. (1994). *Stereocontrolled Organic Synthesis: A Chemistry for the 21th Century Monograph*, edited by B. M. Trost, pp. 337–397. London: Blackwell Scientific.

[bb6] Krief, A., Lorvelec, G. & Jeanmart, S. (2000). *Tetrahedron Lett.* **41**, 3871–3874.

[bb7] Oxford Diffraction (2009). *CrysAlis PRO* Oxford Diffraction Ltd, Yarnton, England.

[bb8] Sheldrick, G. M. (2008). *Acta Cryst.* A**64**, 112–122. 10.1107/S0108767307043930 18156677

[bb9] Spek, A. L. (2009). *Acta Cryst.* D**65**, 148–155. 10.1107/S090744490804362X PMC2631630 19171970

